# Osteoarthritis-like pathologic changes in the knee joint induced by environmental disruption of circadian rhythms is potentiated by a high-fat diet

**DOI:** 10.1038/srep16896

**Published:** 2015-11-20

**Authors:** Ranjan Kc, Xin Li, Christopher B. Forsyth, Robin M. Voigt, Keith C. Summa, Martha Hotz Vitaterna, Beata Tryniszewska, Ali Keshavarzian, Fred W. Turek, Qing-Jun Meng, Hee-Jeong Im

**Affiliations:** 1 Departments of Biochemistry, Rush University Medical Center, Chicago, IL, 60612; 2 Division of Digestive Diseases and Nutrition, Rush University Medical Center, Chicago, IL, 60612; 3 Departments of Orthopaedic Surgery, Internal Medicine, Rush University Medical Center, Chicago, IL, 60612; 4 Center for Sleep and Circadian Biology, Department of Neurobiology, Northwestern University, Evanston, IL, 60208; 5 Sections of Rheumatology, Rush University Medical Center, Chicago, IL, 60612; 6 Departments of Pharmacology, Rush University Medical Center, Chicago, IL, 60612; 7 Departments of Molecular Biophysics and Physiology, Rush University Medical Center, Chicago, IL, 60612; 8 Division of Pharmacology, Utrecht Institute for Pharmaceutical Sciences, Faculty of Science, Utrecht University, Netherlands; 9 Faculty of Life Sciences, University of Manchester, Manchester, United Kingdom, M13 9PT; 10 Department of Bioengineering, University of Illinois at Chicago, Chicago, IL, 60612; 11 Jesse Brown Veterans Affairs Medical Center at Chicago, IL 60612, USA

## Abstract

A variety of environmental factors contribute to progressive development of osteoarthritis (OA). Environmental factors that upset circadian rhythms have been linked to various diseases. Our recent work establishes chronic environmental circadian disruption - analogous to rotating shiftwork-associated disruption of circadian rhythms in humans - as a novel risk factor for the development of OA. Evidence suggests shift workers are prone to obesity and also show altered eating habits (i.e., increased preference for high-fat containing food). In the present study, we investigated the impact of chronic circadian rhythm disruption in combination with a high-fat diet (HFD) on progression of OA in a mouse model. Our study demonstrates that when mice with chronically circadian rhythms were fed a HFD, there was a significant proteoglycan (PG) loss and fibrillation in knee joint as well as increased activation of the expression of the catabolic mediators involved in cartilage homeostasis. Our results, for the first time, provide the evidence that environmental disruption of circadian rhythms plus HFD potentiate OA-like pathological changes in the mouse joints. Thus, our findings may open new perspectives on the interactions of chronic circadian rhythms disruption with diet in the development of OA and may have potential clinical implications.

Osteoarthritis (OA) is characterized by a slow and progressive deterioration of articular cartilage. OA incidence is prevalent in society and is a major cause of disability. Because drivers of OA development appear to be multifactorial, the causative factors responsible for initiation and progression of the disease and the underlying pathogenesis are not yet well understood. Circadian rhythms synchrony in the brain and periphery are necessary for homeostasis. A pleiotropic effect of circadian desynchrony on the musculoskeletal system has only begun to be elucidated. Recently, we have uncovered a link between environmental circadian rhythm disruption - analogous to rotating shiftwork-associated disruption of circadian rhythms in humans - and the induction of OA-like pathology in the mouse knee joints^1^. Specifically, chronic environmental disruption of circadian rhythms predisposes mice to pathological changes in the knee joint as reflected by proteoglycan (PG) loss, fibrillation, upregulation of matrix-degrading enzyme production, and concomitant downregulation of chondrogenic factors. Other recent studies found that circadian clock genes in chondrocytes regulate cartilage homeostasis, supporting a role of circadian dysregulation in the pathogenesis of joint disease^2^.

Circadian rhythms are integral for the normal functioning of numerous physiological processes^3^ and at the molecular level are comprised of an intricate system of positive and negative feedback loops that takes approximately 24 h to complete^4^. Circadian clocks are typically entrained (i.e., set) by daily environmental cues. Under normal circumstances, the master clock in the suprachiasmatic nucleus (SCN) is synchronized to the external environment by exposure to light, and the SCN then entrains the peripheral clocks directly or indirectly found throughout the body. In modern 24/7 societies, the circadian clock is frequently disrupted by excessive night-time exposure to light and physical activities. For example, chronic late-night activity, frequent travel across different time zones and rotational night-time shiftwork are substantial environmental disruptors of normal circadian rhythms. Nearly 15 million Americans work a permanent night shift or regularly rotate in and out of night shifts (Bureau of Labor Statistics), indicating a high prevalence of circadian disruption as a normal part of modern-day society.

Epidemiological evidence suggests that shift workers are more prone to weight gain and are susceptible to obesity-linked adverse health consequences^5^. While an association between obesity and an increased risk of OA has been reported^6^, it is unclear how obesity-linked factors contribute to the pathophysiology of OA. Studies have reported that the eating habits of shift workers are altered^7^,^8^, and there is a higher caloric intake and an increased consumption of foods containing saturated fat^9^,^10^. More importantly, recent studies have demonstrated that a high-fat diet (HFD) can adversely affect circadian rhythms and alter the expression of clock-controlled genes involved in energy homeostasis in mice^11^. Furthermore, a HFD impairs the ability of mice to synchronize their circadian rhythms with the light:dark (LD) cycle, particularly when the LD cycles are repeatedly altered (i.e., a ‘jet-lag’ protocol)^12^. In an effort to better understand the adverse effect of chronic circadian rhythm disruption on OA, in the present study we investigated how HFD interacts with the circadian rhythm disruption protocol to aggravate OA progression.

## Results

### Effect of chronic circadian disruption and a high-fat diet on mouse body weight

**Figure 1:**
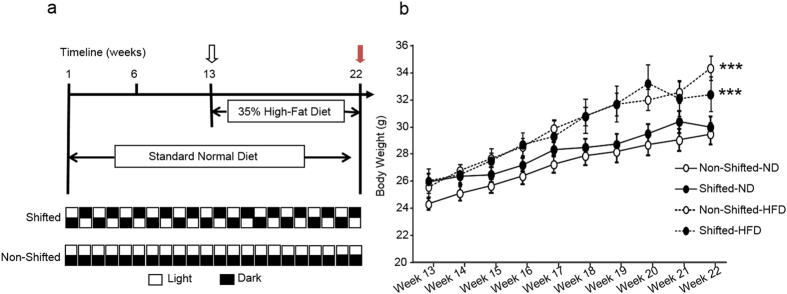
Chronic circadian disruption and high-fat diet increase mouse body weight. (**a**) Experimental protocol for environmental circadian disruption and feeding regimens. (**b**) Mice were weighed once a week and the body weights of mice consuming either the ND or HFD are depicted over the last ten weeks (Week 13 – Week 22) of the experimental period. HFD diet had a significant impact on body weight (***, Diet: *P* = 0.005, Diet x Time Interaction: *P* < 0.0001). There was also a significant circadian rhythm disruption x time interaction on body weight (*P* = 0.046).

The animal’s body weight was monitored throughout the experimental protocol. As expected, the HFD significantly increased body weight (Diet: *P* = 0.005, Diet x Time Interaction: *P* < 0.0001) in both shifted and non-shifted mice (Fig. 1b). Circadian disruption also had effect on body weight (Circadian Disruption: *P* = 0.59, Circadian Disruption x Time Interaction: *P* = 0.046) but the effect was less compared to HFD.

### Chronic circadian disruption and high-fat diet accelerate development of OA in mouse joints

**Figure 2:**
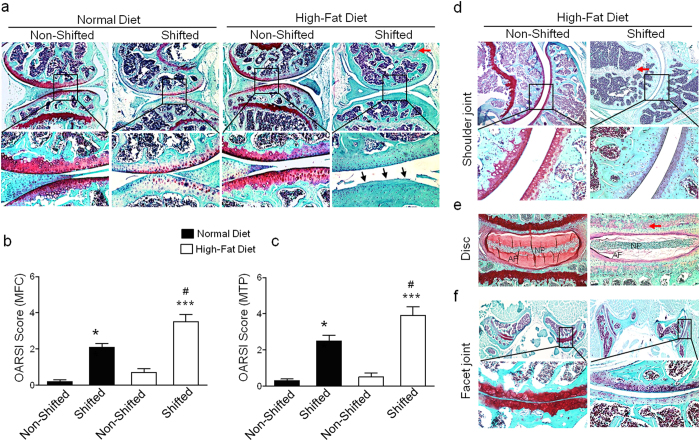
Chronic circadian disruption and high-fat diet accelerate progression of OA in mouse joints. (**a**) Safranin-O/Fast Green staining for knee joint histological evaluation. In (Panel **a**), the upper row of 4 images are at low magnification (4X), while the lower row of 4 images is at higher magnification (20X). Black arrows indicate PG loss and fibrillation. Red arrow indicate PG loss in the growth plate. (**b**,**c**) Severity of articular cartilage degradation was graded using OARSI scoring system. Values are presented as mean ± SD (Comparing non-shifted ND mice to shifted ND mice: **P < 0.01; comparing non-shifted ND mice to shifted HFD mice: ***P < 0.001; comparing shifted ND mice to shifted HFD mice: ^#^*P* < 0.05). MFC, medial femoral condyle; MTP, medial tibial plateau. Histological evaluation for the pathological changes in glenohumeral joints (**d**), lumbar spine intervertebral discs (Magnification: 10X) (**e**) and spine facet joints (**f**) of non-shifted HFD mice and shifted HFD mice by Safranin-O/Fast green staining. In (Panel **d**,**f**), the upper row of 2 images are at low magnification (4X), while the lower row of 2 images is at higher magnification (20X). NP, Nucleus pulposus; AF, Annulus fibrosus. Red arrows indicate PG loss in the growth plate. For each group n = 6.

We next performed histological analyses to evaluate the effects of chronic circadian rhythm disruption and a HFD on the pathological changes in mouse knee joints. Histological examinations of knee joint sections by Safranin-O/Fast Green staining demonstrated significantly reduced PG content with increased fibrillation in the articular cartilage of shifted mice fed a normal diet (ND) compared to the control (non-shifted) mice on a ND. These changes were more pronounced in shifted mice on a HFD compared to control (non-shifted) mice on a HFD: there was dramatic PG loss in articular cartilage as well as in the growth plate (Fig. 2a). In the absence of LD shifting, mice fed a HFD displayed no significant change in PG content in articular cartilage. We next performed semi-quantitative histopathologic grading of Safranin-O/Fast Green-stained knee joint cartilage using a murine scoring system established by Osteoarthritis Research Society International (OARSI) initiative^13^. Histopathological grading was performed in the medial femoral condyle (MFC) and the medial tibial plateau (MTP). In accordance with histological analyses, shifted mice in the ND group had higher OARSI scores for MFC and MTP than non-shifted mice in the ND group (MFC, 2.1 ± 0.20 *vs.* 0.2 ± 0.10; MTP, 2.5 ± 0.20 *vs.* 0.1 ± 0.10; *P* <  0.01). OA severity scores were much higher in shifted mice with the HFD compared to non-shifted with the ND group (MFC, 3.5 ± 0.40 *vs.* 0.2 ± 0.10; MTP, 3.9 ± 0.50 *vs.* 0.1 ± 0.10, *P* < 0.001). Moreover, in the shifted mice, the HFD group had greater OA severity scores than the ND group (MFC, 3.5 ± 0.40 *vs.* 2.1 ± 0.20; MTP, 3.9 ± 0.50 *vs.* 2.5 ± 0.20 *P* < 0.05) (Fig. 2b,c).

Recently, we reported that shifted mice on a ND show OA-like pathological changes exclusively in the knee joints but not in other joints^1^. We next examined whether chronic circadian rhythm disruption and a HFD have an effect on other mouse joints. Interestingly, when shifted mice were put on a HFD, we found pathological changes with significant decrease in PG in several other joints, including the glenohumeral joint (Fig. 2d), lumbar spine intervertebral disc (Fig. 2e) and spine facet joint (Fig. 2f) by Safranin-O/Fast Green staining. In the absence of LD shifting, HFD alone did not show any pathological changes in other joints, indicating that HFD feeding potentiates the progression of OA-like degeneration in several mouse joints with chronic disruption of circadian rhythms.

### Chronic circadian disruption and HFD increase activation of catabolic signaling pathways in the mouse knee joint

**Figure 3:**
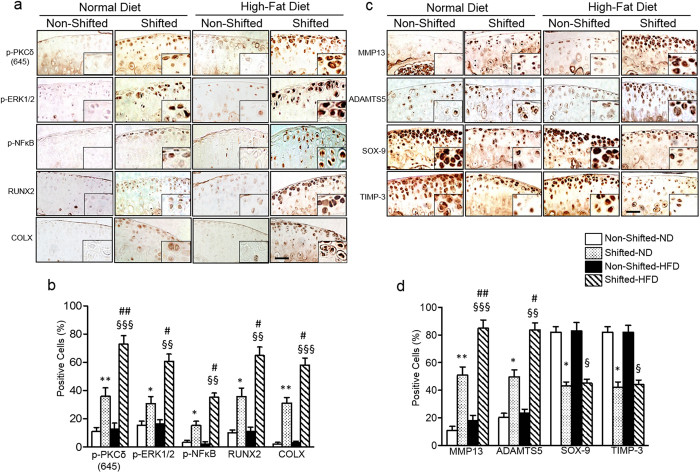
Chronic circadian disruption and HFD increase activation of catabolic signaling pathways in the mouse knee joint. (**a**,**c**) Representative immunohistochemical staining images in knee joint sections. Pictures of immunostaining were obtained under 200X magnification (n = 4 per group), representing medial femoral condyle and tibial plateau. For each immuostain, a negative control without primary antibody was included. Scale bars, 50 μm. (**c**,**d**) Quantitative histomorphometric analyses. Percent of positively stained cells in articular cartilage and calcified cartilage were counted using ImageJ (NIH, Bethesda, MD). For each group n = 4. Values are presented as mean ± SD (Comparing non-shifted ND mice to shifted ND mice: *P < 0.05; **P < 0.01; comparing non-shifted ND mice to shifted HFD mice: ^§§^*P* < 0.01; ^§§§^*P* < 0.001; comparing shifted ND mice to shifted HFD mice: ^#^*P* < 0.05 ^##^*P* < 0.01).

More recently^1^, we demonstrated that articular chondrocytes of environmentally shifted mice show (i) significantly increased activation of the protein kinase Cδ (PKCδ)-ERK-RUNX2/NFκB signaling pathways, (ii) stimulation of matrix-degrading enzymes, matrix metalloprotease-13 (MMP-13) and a disintegrin and metalloproteinase with thrombospondin motifs-5 (ADAMTS-5), as well as (iii) suppression of the anabolic and anti-catabolic mediators, SOX-9 and tissue inhibitor of metalloproteinase-3 (TIMP-3). We, therefore, investigated whether HFD feeding regimens alter the expression of these catabolic and anabolic/anti-catabolic signaling molecules which cause severe pathology in shifted mice with the HFD. Immunohistochemical analyses revealed increased overexpression of PKCδ-ERK-RUNX2/NFκB signaling molecules, chondrocyte hypertrophy marker collagen type-X (ColX) and matrix-degrading enzymes (MMP-13 and ADAMTS-5) in knee joint articular cartilage of shifted mice with the ND compared to the non-shifted group with the ND. Expression levels of these molecules were markedly increased in shifted mice in the HFD group compared to shifted mice in the ND group (Fig. 3a,c; *P* < 0.01). We found that expression levels of chondroprotective molecules (SOX-9 and TIMP-3) were strikingly reduced in shifted mice in the ND group compared to non-shifted mice with the ND (Fig. 3c; *P* < 0.05). However, a HFD did not further reduce the expression levels of SOX-9 and TIMP-3 in knee joints of shifted mice compared to shifted mice with the ND. We quantified these findings by histomorphometric analyses (Fig. 3b,d). As expected, HFD alone did not cause any significant changes in catabolic and anabolic signaling molecules in articular cartilage of knee joint with non-shifted mice. These findings clearly demonstrate that HFD accentuates OA pathology in knee joints of circadian rhythm disrupted mice.

## Discussion

The results of the present study demonstrate that chronic circadian rhythm disruption in combination with a HFD potentiates progression of OA in mouse joints. Using an established *in vivo* model, we found that chronic disruption of circadian rhythms plus HFD led to significant PG loss and fibrillation in the knee joints of mice and showed increased expression of catabolic mediators involved in cartilage homeostasis. Additionally, HFD also induced pathological changes in several other joints including the glenohumeral joint, lumbar spine intervertebral disc and spine facet joint of chronically circadian rhythms disrupted mice. Our study is the first to show that chronic circadian disruption plus HFD potentiate OA and emphasize their substantial influence on the musculoskeletal system.

Chronic circadian disruption, achieved with repeated exposure to phase shifts of the LD cycle, has been shown to accelerate and/or exacerbate numerous pathologies in rodents^14^,^15^. Our recent study demonstrated that chronic disruption of circadian rhythms induces OA-like pathological changes in mice knee joints by suppressing proteoglycan accumulation, up-regulating matrix-degrading enzymes and down-regulating anabolic mediators. In the present study we found that shifted mice when fed with a HFD diet showed increased OA-like features compared to shifted mice fed a ND. Moreover, catabolic signaling pathways PKCδ-ERK-RUNX2/NFκB, chondrocyte hypertrophy marker (ColX) and matrix-degrading enzymes (MMP-13 and ADAMTS-5) were significantly increased in knee joint articular cartilage of shifted mice with a HFD compared to the shifted mice with the ND indicating that HFD diet accentuates deleterious effects of circadian disruption in mouse knee joint pathology. Our current findings coincide with the notion that the adverse effects of chronic circadian disruption become more apparent if the animal is challenged with second physiological insult. For example, our group recently showed that when challenged with an alcohol-containing diet, mice in chronic phase shifts in the LD cycle resulted in increased gut leakiness^16^. Similarly, when mice are challenged with dextran sodium sulfate (DSS), chronic LD cycle shift mice showed more severe colonic inflammation and overall poor health^14^. Indeed, our shifted mice when challenged by a HFD showed severe OA pathology compared to shifted mice in ND. Our results show that shifted mice in HFD showed severe OA-like pathological changes not only in the knee joint but also in several other joints. Previous studies have also demonstrated that HFD can accelerate the progression of OA after a defined OA initiation^17^,^18^. Recently, we reported that chronic disruption of circadian rhythms initiates OA-like pathological changes specifically in the mouse knee joint. Thus, in the present study, severe OA-like pathological changes seen in several joints could be due to synergistic detrimental effects of chronic circadian rhythm disruption and HFD.

Our results show that HFD in combination with chronic circadian disruption had a significant effect on the pathology of mouse joints. Mice fed HFD alone did not show significant pathological change in knee articular cartilage as well as in the other joints. These findings resonate with previous findings by Robert and colleagues^17^ who similarly reported that HFD alone had little effect on OA pathology in the mouse knee joint. Several studies have previously reported a relationship between HFD alone and induction of OA, but with conflicting results^17^,^19^,^20^. Moreover, in previous studies use of HFD in OA studies varied from moderate 32 kcal% to very high 60 kcal% fat^17^,^19^. From a nutritional perspective, the dietary HF content of 30 kcal% is considered as moderate and more human relevant fat content^21^. Therefore, in this study, we used moderate HFD (35% of total daily caloric intake) to model more common high fat Western type diet rather than very high fat diet, which is not commonly consumed. Furthermore, since the primary goal of this study was to determine whether a HFD exacerbates the negative impact of disrupted circadian rhythm on joints, we wanted to avoid a potential “ceiling effect” because prior reports indicate that diets very high in fat promote OA in animal models^20^. It is known that HFD feeding can induce metabolic abnormalites^22^. Moreover, circadian disorganization also induces metabolic disorders and appears to promote deleterious health consequences^23^,^24^. Since metabolic disorders are associated with OA and our shifted mice in HFD showed severe OA pathology, we suggest that deleterious effects of circadian disruption and a HFD synergize to activate OA promoting pathways. However, further studies are warranted to more fully elucidate mechanisms that underlie the deleterious effects of the simultaneous chronic circadian disruption and HFD on the degenerative process in joint tissues.

Finally, there are several limitations of this study that must be taken into account. First, we have shown the effects of chronic circadian disruption and HFD in an *in vivo* mouse model. Since nearly 15% of the US working population engages in some sort of shift work, loosely defined to include static night shifts, flex shifts, extended shifts, rotating shifts, and frequent international travel by airline flight crews, it is worth investigating whether the observations found in mice apply to humans, i.e., whether shift workers with unhealthy diet are at higher risk of developing OA. Second, our results suggest that, at least in part, the PKCδ-ERK-NFκB and Runx2 signaling cascades in the cartilage are involved in the accelerated OA progression. Further studies on comprehensive cell-signaling pathways and molecular mechanisms underlying these findings remain to be elucidated. Third, the metabolic effects of the HFD and chronic circadian rhythm disruption were not evaluated in this study. Therefore, the combined metabolic effects of the HFD and disrupted circadian rhythms and their relationship to OA should be evaluated in the future studies.

## Conclusion

Our results, for the first time, demonstrate that simultaneous chronic circadian disruption and HFD feeding substantially contribute to exacerbating OA-like pathology in the mouse joints. Our findings open new perspectives on chronic circadian rhythm disruption plus HFD in the development of OA, and may have implications for devising novel strategies for OA treatment in the future.

## Materials and Methods

### Animals and Circadian Rhythms disruption

In our experimental protocol, young adult (6–8 weeks old) WT C57BL/6J male mice obtained from Jackson Laboratory (Bar Harbor, ME) were housed individually in cages stored within ventilated, light-tight cabinets under a standard 12 hour light:12 hour dark cycle. As shown in Fig. 1a, mice were randomly divided into one of two groups: (1) the non-shifted group (n = 20) which was maintained on a fixed LD cycle or (2) the shifted group (n = 20), which underwent reversals of the 24 h LD cycle at the end of each week. These circadian protocols were maintained for the duration of the experiment (i.e., 22 weeks). All mice were housed and handled in accordance with federal animal welfare guidelines and in compliance with the Public Health Service Policy on Humane Care and Use of Laboratory Animals (2002) and the Guide for the Use and Care of Laboratory Animals (8^th^ Edition). All experimental protocols were reviewed and approved by the Institutional Animal Care and Use Committee of Rush University Medical Center.

### Feeding Protocol

For the feeding protocol, all mice were maintained on a standard normal diet (ND) for the first 12 weeks of the study (Harlan Teklad Global 18% Protein Rodent Diet; 18.6% protein, 44.2% carbohydrate, 6.2% fat, 3.5% crude fiber). After 12 weeks, mice were either placed on a HFD (36% protein, 29% carbohydrate (dextrose), and 35% fat) or continued on a ND for an additional 10 weeks while being maintained on the same stable of weekly-shifted LD cycle. The diet was freshly prepared each day and supplied to mice in graduated sipper tubes (Bio-Serv, Frenchtown, NJ). All animals were allowed unrestricted activity and were provided food and water *ad libitum*.

### Histology and Immunohistochemistry Analyses

Upon completion of the experiment all mice were euthanized, knee joints were dissected aseptically and fixed in 4% paraformalin, decalcified and paraffin-embedded. Knee joints were serially sectioned in a sagittal plane, and 3-4 representative midsagittal 7-μm-thick sections were selected and stained with Safranin-O/Fast Green for histological evaluation. OA grade was determined using the OARSI scoring system^13^. Immunohistochemical staining was performed using the standard avidin-biotin-peroxidase complex technique. Sections were then visualized using Vectastain Kit (Vector Laboratories). For each immuostain, a negative control without primary antibody was included. Immunostained pictures were obtained under 200X magnification (n = 5 per group), representing medial femoral condyle and tibial plateau and then the percent of positively stained cells in articular cartilage and calcified cartilage were counted using ImageJ (NIH, Bethesda, MD). Two different investigators blinded to the study performed all histomorphometric analyses.

### Reagents

Antibodies used were phospho-PKCδ (Ser645), MMP-13, SOX-9, Collagen Type-X (Millipore, Billerica, MA), ADAMTS-5, (Thermo Fisher Scientific, Rockford, IL), RUNX-2, TIMP-3 (Abcam, Cambridge, MA), phospho-ERK1/2(Thr202/Tyr204), phospho-NF-κB-p65(Ser536) (Cell Signaling Technology, Danvers, MA).

### Statistical Analysis

Statistical significance was determined by Student’s t-test or analysis of variance for repeated measures, followed by step-down Bonferroni’s multiple comparison post-test, as appropriate, using SPSS 17 software (IBM Corporation). Body weight was evaluated via three-way repeated measures analysis of variance (ANOVA) using GB-STAT version 10.0 (Dynamic Microsystems, Silver Spring, MD). *P* values lower than 0.05 were considered to be statistically significant.

## Additional Information

**How to cite this article**: Kc, R. *et al.* Osteoarthritis-like pathologic changes in the knee joint induced by environmental disruption of circadian rhythms is potentiated by a high-fat diet. *Sci. Rep.* **5**, 16896; doi: 10.1038/srep16896 (2015).

## References

[b1] Kc R. *et al.* Environmental disruption of circadian rhythm predisposes mice to osteoarthritis-like changes in knee joint. Journal of cellular physiology 230, 2174–2183 (2015). 25655021 10.1002/jcp.24946 PMC4447623

[b2] Gossan N. *et al.* The circadian clock in murine chondrocytes regulates genes controlling key aspects of cartilage homeostasis. Arthritis and rheumatism 65, 2334–2345 (2013). 23896777 10.1002/art.38035 PMC3888512

[b3] Reppert S. M. & Weaver D. R. Coordination of circadian timing in mammals. Nature 418, 935–941 (2002). 12198538 10.1038/nature00965

[b4] Reppert S. M. & Weaver D. R. Molecular analysis of mammalian circadian rhythms. Annual review of physiology 63, 647–676 (2001). 10.1146/annurev.physiol.63.1.647 11181971

[b5] Kubo T. *et al.* Retrospective cohort study of the risk of obesity among shift workers: findings from the Industry-based Shift Workers’ Health study, Japan. Occupational and environmental medicine 68, 327–331 (2011). 20884794 10.1136/oem.2009.054445

[b6] Wluka A. E., Lombard C. B. & Cicuttini F. M. Tackling obesity in knee osteoarthritis. Nature reviews Rheumatology 9, 225–235 (2013). 23247649 10.1038/nrrheum.2012.224

[b7] Waterhouse J., Buckley P., Edwards B. & Reilly T. Measurement of, and some reasons for, differences in eating habits between night and day workers. Chronobiology international 20, 1075–1092 (2003). 14680144 10.1081/cbi-120025536

[b8] Pasqua I. C. & Moreno C. R. The nutritional status and eating habits of shift workers: a chronobiological approach. Chronobiology international 21, 949–960 (2004). 15646241 10.1081/cbi-200040310

[b9] Cain S. W., Filtness A. J., Phillips C. L. & Anderson C. Enhanced preference for high-fat foods following a simulated night shift. Scandinavian journal of work, environment & health 41, 288–293 (2015). 10.5271/sjweh.3486 25699635

[b10] Di Lorenzo L. *et al.* Effect of shift work on body mass index: results of a study performed in 319 glucose-tolerant men working in a Southern Italian industry. International journal of obesity and related metabolic disorders: journal of the International Association for the Study of Obesity 27, 1353–1358 (2003). 10.1038/sj.ijo.0802419 14574346

[b11] Kohsaka A. *et al.* High-fat diet disrupts behavioral and molecular circadian rhythms in mice. Cell metabolism 6, 414–421 (2007). 17983587 10.1016/j.cmet.2007.09.006

[b12] Mendoza J., Pevet P. & Challet E. High-fat feeding alters the clock synchronization to light. The Journal of physiology 586, 5901–5910 (2008). 18936083 10.1113/jphysiol.2008.159566 PMC2655413

[b13] Glasson S. S., Chambers M. G., Van Den Berg W. B. & Little C. B. The OARSI histopathology initiative - recommendations for histological assessments of osteoarthritis in the mouse. Osteoarthritis and cartilage/OARS, Osteoarthritis Research Society 18 Suppl 3, S17–23 (2010). 10.1016/j.joca.2010.05.025 20864019

[b14] Preuss F. *et al.* Adverse effects of chronic circadian desynchronization in animals in a “challenging” environment. American journal of physiology Regulatory, integrative and comparative physiology 295, R2034–2040 (2008). 10.1152/ajpregu.00118.2008 PMC2685296 18843092

[b15] Castanon-Cervantes O. *et al.* Dysregulation of inflammatory responses by chronic circadian disruption. Journal of immunology 185, 5796–5805 (2010). 10.4049/jimmunol.1001026 PMC2974025 20944004

[b16] Summa K. C. *et al.* Disruption of the Circadian Clock in Mice Increases Intestinal Permeability and Promotes Alcohol-Induced Hepatic Pathology and Inflammation. PloS one 8, e67102 (2013). 23825629 10.1371/journal.pone.0067102 PMC3688973

[b17] Mooney R. A., Sampson E. R., Lerea J., Rosier R. N. & Zuscik M. J. High-fat diet accelerates progression of osteoarthritis after meniscal/ligamentous injury. Arthritis research & therapy 13, R198 (2011). 22152451 10.1186/ar3529 PMC3334649

[b18] Louer C. R. *et al.* Diet-induced obesity significantly increases the severity of posttraumatic arthritis in mice. Arthritis and rheumatism 64, 3220–3230 (2012). 22576842 10.1002/art.34533 PMC3426642

[b19] Iwata M. *et al.* Initial responses of articular tissues in a murine high-fat diet-induced osteoarthritis model: pivotal role of the IPFP as a cytokine fountain. PloS one 8, e60706 (2013). 23593289 10.1371/journal.pone.0060706 PMC3625196

[b20] Griffin T. M., Huebner J. L., Kraus V. B., Yan Z. & Guilak F. Induction of osteoarthritis and metabolic inflammation by a very high-fat diet in mice: effects of short-term exercise. Arthritis and rheumatism 64, 443–453 (2012). 21953366 10.1002/art.33332 PMC3268860

[b21] van Schothorst E. M., Bunschoten A., Verlinde E., Schrauwen P. & Keijer J. Glycemic index differences of high-fat diets modulate primarily lipid metabolism in murine adipose tissue. Physiological genomics 43, 942–949 (2011). 21673076 10.1152/physiolgenomics.00042.2011

[b22] Buettner R., Scholmerich J. & Bollheimer L. C. High-fat diets: modeling the metabolic disorders of human obesity in rodents. Obesity 15, 798–808 (2007). 17426312 10.1038/oby.2007.608

[b23] Turek F. W. *et al.* Obesity and metabolic syndrome in circadian Clock mutant mice. Science 308, 1043–1045 (2005). 15845877 10.1126/science.1108750 PMC3764501

[b24] Morris C. J., Yang J. N. & Scheer F. A. The impact of the circadian timing system on cardiovascular and metabolic function. Progress in brain research 199, 337–358 (2012). 22877674 10.1016/B978-0-444-59427-3.00019-8 PMC3704149

